# What's New in the Treatment of Systemic Lupus Erythematosus

**DOI:** 10.3389/fmed.2021.655100

**Published:** 2021-03-05

**Authors:** Stamatis Nick Liossis, Chrysanthi Staveri

**Affiliations:** ^1^Division of Rheumatology, Department of Internal Medicine, Patras University Hospital, Patras, Greece; ^2^Division of Rheumatology, Department of Internal Medicine, University of Patras Medical School, Patras, Greece

**Keywords:** systemic lupus erythematosus, treatment, clinical trial, B cell, lupus nephritis

## Abstract

Systemic lupus erythematosus (SLE) is a chronic autoimmune multisystem disease with a variable presentation and manifestations ranging from mild to severe or even life-threatening. There is an ongoing and unmet need for novel, disease-specific, effective and safe treatment modalities. The aim of this review is to summarize data on SLE treatment that have emerged over the last 3 years. We will put emphasis on studies evaluating potential treatments on severe lupus manifestations such as lupus nephritis. Despite the existence of several therapeutic agents in SLE, the disease keeps causing significant morbidity. It is encouraging that a variety of therapeutic options are currently under investigation, although there are occasional trial failures.

## Introduction

Systemic lupus erythematosus (SLE) is an astonishing heterogeneous multisystem autoimmune disease with a quite unpredictable outcome. Patients suffering from SLE are typically treated with corticosteroids and immunosuppressive agents (1). An eminent direct or indirect target of novel therapeutic approaches has been the lupus B cell (2–4). Among them, only belimumab that inhibits B cell survival has been approved for patients with SLE and SLE-related nephritis. Rituximab (RTX) causing B cell depletion can also be administered according to the ACR and EULAR guidelines in refractory lupus nephritis despite failed clinical trials, and is often used off-label for other manifestations as well, based on the encouraging results of diverse studies. This reflects one of the problems of failed clinical trials in patients with SLE: failure to suppress one specific SLE manifestation, such as lupus nephritis, may not exclude encouraging outcomes for some other aspects of the disease, such as hematological, mucocutaneous, or articular involvement. Inadequate control of lupus nephritis may potentially result to end-stage renal disease due to irreversible damage of the kidneys. Measurement of proteinuria is a useful tool to assess disease activity in patients with kidney involvement and an early renal response is judged by a decrease of proteinuria; improvement of proteinuria at 12 months of treatment correlates well with a favorable long-term renal outcome. Despite progress, a complete renal response is not achieved in more than 40% of patients with lupus nephritis. Other manifestations are also commonly less-than-satisfactorily treated. Therefore, additional and new approaches are being evaluated.

## The Cellular Approach

The B cell, as a major component of the adaptive immune system, may mediate autoimmune disease. B cells are not only capable of producing autoantibodies after their differentiation into plasma cells, but they also present autoantigens to T cells and they secrete cytokines. Therefore, B cells represent an established and clear target of treatment approaches; lupus B cells have been targeted either directly via regimens that cause B cell depletion or indirectly via regimens affecting B cell survival, or via inhibiting their antigen-receptor-initiated function.

### Killing the B Cell

The B cell has been targeted in SLE since decades. Initially considered guilty only as autoAb producers, B cells were subsequently also recognized as efficient antigen-presenting cells and cytokine producers. Works from the Craft Lab disclosed that murine lupus could indeed develop in T cell deficient animals (5). In contrast, it was principally with the works of Chan et al. that a central, eminent, and indispensable pathogenetic role was assigned to the B cell in murine lupus models (6, 7). In humans, critical functions of the B cell, such as the antigen-receptor initiated activation was revealed to be intrinsically abnormal (Liossis et al., work from the Tsokos Lab) (2). Anolik and Leandro from the Departments of Looney and Isenberg, respectively, were the first to administer the B cell depleting mAb RTX in a few patients with SLE with promising results (8, 9).

Obinutuzumab, a type II humanized anti-CD20 monoclonal antibody (mAb) that depletes B cells has been tested in patients with lupus nephritis presenting some very encouraging results. More than 100 patients with Class III or Class IV lupus nephritis were randomized to obinutuzumab or placebo given along with corticosteroids and mycophenolate mofetil (MMF) (10). The primary end point was complete renal response at week 52. Complete renal response was achieved in 40% of the patients in the obinutuzumab group and in 18% of the patients in the placebo group at week 76 (*p* = 0.007); this favorable response was sustained in 41% of the patients in the obinutuzumab group and in 23% of the patients in the placebo group through week 104 (*p* = 0.026). Serious infections were recorded in 8% in the obinutuzumab group and in 18% in the placebo group, making obinutuzumab not only an efficacious but also a safe choice in the management of lupus nephritis. Flow cytometry measurements at weeks 24 and 52 of obinutuzumab treatment were employed to assess sustained B cell depletion (11). Obinutuzumab resulted in a remarkable B cell depletion as early as 4 weeks after obinutuzumab treatment. Patients that achieved sustained B cell depletion, according to the flow cytometry measurements at weeks 24 and 52, had a more favorable outcome of their renal disease at week 76, emphasizing the importance of B cell depletion in the disease progress.

### Alternative Sequencing of Biologics in SLE

Another study assessed the efficacy of switching RTX to other, alternative anti-CD20 agents in comparison to switching to belimumab in SLE patients who had a secondary failure to RTX (12). Secondary failure was reported in patients initially responding (and depleting B cells) that subsequently developed serious infusion reactions, or did not sustain B cell depletion, or failed to sustain a good clinical response. One hundred and twenty-five patients were treated with RTX and 14 of them had a secondary failure. Eight out of these 14 patients were switched to belimumab and 6/14 patients were switched to an alternative humanized anti-CD20 agent. More specifically, ocrelizumab was substituted in 3 patients, ofatumumab was administered in 2 patients and obinutuzumab was substituted in 1 patient. In the belimumab group, a new or worsening British Isles Lupus Assessment Group (BILAG)-2004 grade A for lupus nephritis was noticed in 2 patients, whereas SLEDAI-2K scores yielded disappointing results. Additionally, the median required dose of prednisone was increased from 7.5 mg at baseline to 10 mg at 6 months. In contrast, in the second group, all 6 patients achieved an SLE Responder Index (SRI)-4 response. Median SLEDAI-2K improved from 16 at baseline to 5 at 6 months. The median dose of prednisone was reduced from 15 to 10.5 mg. In conclusion, switching to alternative humanized anti-CD20 mAb could be considered in SLE patients with secondary failure to RTX, instead of replacing the B cell depletion approach with belimumab treatment. Belimumab was capable of sustaining a good response following daratumumab-mediated plasma cell depletion treatment (as discussed in Plasma Cells) but seems to lack efficacy to sustain remission following B cell depletion.

### Silencing (Instead of Killing) the B Cell

Obexelimab is a mAb that targets the CD19 molecule expressed on the surface of B cells. However, obexelimab simultaneously binds the Fcγ receptor IIb (FcγRIIb) the only inhibitory Fcγ receptor that is also expressed on the surface of B cells. Therefore, obexelimab inhibits the activation of B cells without depleting them. In a phase II study, 104 patients were randomly assigned to receive obexelimab or placebo after achieving low disease activity by intramuscular (IM) steroids and after discontinuing previous immunosuppression (13). Maintenance of improvement was observed through day 225 in 42% of patients in the obexelimab group and in 28.6% of patients in the placebo group (*p* = 0.18). Nevertheless, patients in the obexelimab group showed a significantly longer time to loss-of-improvement (median: 230 vs. 131 days for patients in the placebo group, *p* = 0.025). Remarkably, a group of patients displaying a quite decreased risk of flare during obexelimab treatment has been recently identified (14). In this subgroup of patients, evaluation of gene expression by RNA-sequencing showed that CD27 was the dominant biomarker, followed by other T-cell genes such as CD28 and TCF7. Even though obexelimab targets B but not T cells, these findings suggest that T cells, directly or indirectly, guide obexelimab results.

### Targeting the T Cell

T cells also play a critical role in the pathogenesis of SLE. Belatacept is a fusion protein consisting of the Fc segment of the human IgG1 immunoglobulin and the extracellular domain of CTLA-4. Therefore, belatacept is a costimulation blocker; by blocking the B7-CD28 interaction it selectively inhibits T-cell activation. A retrospective study evaluated the efficacy of belatacept administered in lupus nephritis of 6 patients following renal transplantation (15). Five patients had stable creatinine levels over the following 6 months after belatacept treatment, one patient returned to hemodialysis and another patient was re-listed for a kidney transplant. Mean SLEDAI-2K decreased from 13 to 7.6 in 3 patients. An improvement of extrarenal manifestations along with a stabilization of allograft function are proposed to ensure the beneficial effects of this agent, despite the enrollment of a small number of patients.

Lulizumab is a mAb against CD28, the T cell costimulatory molecule that is essential for T cell activation. In a phase II 24-week study, lulizumab was administered at a dose of 12.5 mg/week or at doses of 1.25 and 5 mg, 12.5 every other week or placebo in combination with standard treatment in 349 patients with SLE (16). Measurement tools of disease activity such as the British Isles Lupus Assessment Group Based Composite Lupus Assessment (BICLA) response rate, CLASI (Cutaneous Lupus Erythematosus Disease Area and Severity Index), and SLEDAI (Systemic Lupus Erythematosus Disease Activity Index) did not show any significant changes between groups.

Rigerimod or Lupuzor (IPP-201101) is a peptide, a fragment of the small nuclear ribonucleoprotein U1-70K. It is thought to act as an immunomodulator by binding major histocompatibility complex (MHC) class II and hence inhibiting T-cell reactivity, leading to a partial restoration of immune tolerance. In a phase III study, it was given subcutaneously at a dose of 200 mg every 4 weeks for 48 weeks in addition to standard treatment (17). A small non-significantly better response rate was noticed over placebo (52.5 vs. 44.6%, *p* = 0.26). Based on the above it is clear that such approaches that target the T cells were more-or-less ineffective. Costimulation blockade has not been rewarding in the treatment of patients with SLE, pointing perhaps to other-than-this pathway targets.

### Plasma Cells

Daratumumab, a mAb approved for the treatment of multiple myeloma, is an IgG1k mAb directed against CD38 causing depletion of plasma cells. Long-lived plasma cells are residents in niches in the bone marrow or (perhaps more importantly) in inflamed tissue and they do not respond to immunosuppressants, including B-cell-targeting treatments. Two patients with severe manifestations of SLE received daratumumab at a dose of 16 mg/kg of body weight once a week for 4 weeks followed by maintenance treatment with I.V. belimumab (18). Daratumumab treatment resulted in remarkable clinical outcomes not only of severe manifestations such as lupus nephritis, autoimmune hemolytic anemia and autoimmune thrombocytopenia but also on less severe manifestations such as arthritis, skin rashes, pericarditis, cutaneous vasculitis, alopecia, and mucosal ulcers. Daratumumab treatment was also associated with favorable serologic responses. Importantly, previous therapeutic interventions with a variety of agents such as bortezomib, mycophenolate mofetil, and cyclophosphamide were ineffective. Despite the extremely small number of patients, data are encouraging supporting further evaluation of daratumumab in meaningfully larger numbers of patients with SLE. It is of interest however that the authors did not ascribe their anti-CD38 mAb-mediated clinical effect(s) exclusively to reductions of plasma cell numbers. Other circulating cells also express CD38 and their numbers decreased following daratumumab treatment. Among them are subsets of B cells, plasmacytoid dendritic cells, and a greatly expanded CD38^+^ T cell subpopulation. Only recently it was shown by Katsuyama et al. that this expanded CD38^+^CD8^+^ T cell subset is responsible for the significantly compromised cytotoxicity encountered in patients with lupus (19).

### Plasmacytoid Dendritic Cells

Type 1 interferons, currently thought of as central to SLE pathogenesis, are secreted in abundance by plasmacytoid dendritic cells (pDCs) when activated.

VIB7734 is a mAb that binds to ILT7, a surface molecule of pDCs, resulting in their elimination and furthermore the reduction of other cytokines such as TNF-α and IL-6. A phase 1, randomized, double-blind, placebo-controlled study assessed VIB7734 in 3 cohorts (20). Cohort 1 included 6 patients with SLE or Sjögren's syndrome without an active disease necessarily. Cohorts 2 and 3 included patients with SLE or cutaneous lupus erythematosus (CLE) with a CLE Disease Area and Severity Index Activity score (CLASI-A) ≥8. The median change of CLASI-A from baseline to month 3 was −5 in the 50 mg group, −9.5 in the 150 mg group, and −5 in the placebo group. In addition, a ≥50% improvement in CLASI-A was achieved in 56% of the patients treated with VIB7734 and in 29% of the patients in the placebo group at month 3. Treatment with VIB7734 was generally safe.

BIIB059 is a humanized IgG1 mAb that binds the specific receptor of pDC BDCA2 (blood dendritic cell antigen 2), and inhibits the production of IFN-I. A 2-part phase II study evaluated the effects of BIIB059 in patients with SLE (part A) and in patients with CLE (part B) (21). The study succeeded to meet its primary endpoint which was the change in total inflamed joints (swollen and tender joints) between baseline and week 24. Total active joint count significantly decreased in the BIIB059 450 mg group [−15.0 vs. −11.6 in the placebo group (*p* = 0.037)]. A numerically increased CLASI-50 response was observed in the BIIB059 group vs. placebo without reaching statistical significance. Adverse events were noticed in 67.9% in the placebo group and 59.2% in the BIIB059 group. A further evaluation of part B demonstrated a statistically significant change of CLASI-A score from baseline to week 16 (19, 22). BIIB059 was highlighted as an alternative therapeutic option in patients with SLE and cutaneous manifestations; however, patients with severe lupus manifestations were not enrolled in this study. Studies that have adopted “The Cellular Approach” can be seen in Table 1.

**Table 1:** Cells targeted in the treatment of lupus.

**Drug**	**Mechanism of action**	**Phase of the study**	**Manifestation**	**Primary outcome**
Obinutuzumab	Anti-CD20 mAb: causes B cell depletion	II	Lupus nephritis	Complete renal response at week 52
Obexelimab	Anti-CD19 mAb: inhibits B cell activation	II	SLE	Loss of improvement at day 225
Belatacept	CTLA4-IgG1: blocks T cell costimulation	Retrospective	Lupus nephritis following renal transplantation	
Lulizumab	Anti-CD28 mAb: inhibits T cell activation	II	SLE	Proportion of responders using BILAG-BICLA at day 169
Rigerimod or Lupuzor	Peptide-fragment of the small ribonucleoprotein U1-70K: Inhibits T cell reactivity through binding to MHC class II	III	SLE	SRI response at week 52
Daratumumab	Anti-CD38 mAb: causes plasma cell depletion	Case report	SLE and lupus nephritis	
VIB7734	mAb: binds to ILT7 of pDCs resulting in their elimination	I	SLE Sjogren's and CLE	
BIIB059	mAb: binds to BDCA2 receptor of pDCs, inhibiting thereby the production of IFN-I	II	SLE (part A) CLE (part B)	Change in total active joint count from baseline to week 24 CLASI-A at week 16

## The Cytokines Approach

### Inhibition of BLyS

From discovery in experimental animals to availability for everyday clinical practice, the story of BLyS/BAFF and anti-BLyS mAb is unprecedented (23, 24). Following BLyS description, its role in human autoimmunity was sought; circulating levels of BLyS are elevated in patients with SLE as described by the group of Stohl, and therefore it was targeted therapeutically (25).

#### Blocking BLyS in Lupus Nephritis

The potential effects of belimumab in lupus nephritis specifically were not known, because the large clinical trials leading to the approval of belimumab, the specific BLyS (B lymphocyte stimulator)-inhibitor, had excluded patients with severe lupus nephritis. Additionally, we previously reported two patients in which lupus nephritis manifested shortly after the initiation of belimumab treatment (26). Of notice, both these patients improved immediately by withdrawal of belimumab and before the initiation of standard therapy. Furthermore, a retrospective study recently reported that introducing belimumab into a standard treatment regimen of patients with lupus without nephritis resulted in development of lupus nephritis with an increased frequency compared to a control group of patients with lupus (hazard ratio, HR: 10.7; *p* = 0.012) (27). The authors proposed that concomitant treatment with antimalarials was protective over this “nephritogenic” potential of belimumab (HR: 0.2; *p* = 0.046).

To formally address the question of its efficacy and safety in lupus nephritis, an international phase III, 104-week, randomized, double-blind, placebo-controlled trial of intravenous (IV) belimumab (BLISS-LN) in addition to standard treatment was recently completed (28). A total of 448 patients were randomized to receive belimumab or placebo (1:1). The primary end point was the primary efficacy renal response at week 104, an endpoint that excluded partial renal response and was defined as an urinary protein to creatinine ratio (UPCR) of 0.7 or less, an estimated glomerular filtration rate (eGFR) that had not declined more than 20% below the levels before the flare or was >60 ml/min/1.73 m^2^ and no use of rescue therapy in cases of treatment failure. Primary efficacy renal response was noticed in 43% of the patients that were treated with belimumab given on top of standard treatment and in 32% of the patients that were treated with placebo in combination with standard treatment (*p* = 0.03) at week 104. Complete renal response at week 104 was one of the major secondary end points and was defined as an UPCR of <0.5, an eGFR that did not decline more than 10% below the levels before the flare, or was >90 ml/min/ 1.73 m^2^ and no use of rescue treatment in cases of therapy failure. More patients in the belimumab group compared to the placebo group had a complete renal response at week 104 (30 vs. 20%; *p* = 0.02). The risk of death or a renal-associated event was also a secondary end point and was significantly lower in the belimumab group compared to the placebo group (HR: 0.51, *p* = 0.001). Regarding safety, no differences were recorded between the two groups of patients. Consequently, the addition of belimumab on top of standard of care may work better in patients with lupus nephritis without particular concerns regarding safety. Although a significant number of patients with lupus nephritis was enrolled in each arm of the study, no subgroups of the patients that might benefit the most from belimumab treatment were identified. In addition, although a better outcome was recorded in 11% more patients, the percentages of responding patients are still far from impressive. The FDA recently approved intravenous belimumab for the treatment of patients with lupus nephritis.

#### Firstly Kill B Cells and Then Inhibit BLyS to Sustain Depletion

B cell depletion following RTX treatment is associated with a sharp homeostatic rise of circulating levels of BLyS. Therefore, treatment at the time when circulating BLyS peaks with belimumab might seem like a rational approach not only to sustain depletion but also to avoid B cell population reconstitution as well. The autoimmune B cell subpopulation might be more sensitive to belimumab-mediated BLyS inhibition. A phase II trial assessed the effect of induction therapy with RTX followed by maintenance therapy with belimumab in 43 patients with recurrent or refractory lupus nephritis (29). Of these, 21 patients received rituximab, cyclophosphamide and glucocorticoids and subsequently weekly belimumab infusions until week 48 and 22 patients received rituximab and cyclophosphamide without belimumab infusions. Complete renal response was defined as an UPCR <0.5, an eGFR ≥120 ml/min/1.73 m^2^, or >80% improvement if eGFR was <120 ml/min/1.73 m^2^ at baseline. Partial renal response was defined as >50% improvement of the UPCR at baseline. Total and circulating autoreactive B cells were measured by flow cytometry. Renal response (complete or partial) was achieved in 52% of the patients in the belimumab group and in 41% of the patients that did not receive belimumab (*p* = 0.452) at week 48. At least one serious infectious adverse event of grade 3 or higher (according to the National Cancer Institute Common Terminology Criteria for Adverse Events) was noticed in 23% of the patients that did not receive belimumab and in 9.5% of the patients in the belimumab group. Sequential therapy with belimumab was generally safe but it does not seem to improve significantly lupus nephritis. This unfavorable clinical response was in contrast to a good and well-sustained B cell depletion profile in the belimumab group. Moreover, the autoreactive B cells were indeed significantly suppressed, despite the disparity in clinical outcomes.

### Inhibition of (Other Than BLyS) B Cell Survival Signals

Telitacicept (RC18) is a novel recombinant TACI-Fc (transmembrane activator and calcium modulator and cyclophilin ligand interactor) fusion protein that binds to soluble BLyS and APRIL (A proliferation inducing ligand) prohibiting thus their biological activities, that go beyond the B cells and affect the plasma cells as well. Therefore, telitacicept inhibits the development and survival of mature B cells and plasma cells without affecting early and memory B cells. In a phase 2b study, patients with a Safety of Estrogen in Lupus Erythematosus National Assessment (SELENA)-SLEDAI score ≥8, consistent with active disease, received telitacicept at doses of 80, 160, and 240 mg or placebo along with standard treatment (30). The primary endpoint was an SRI-4 at week 48. An SRI-4 was achieved in 71.0, 68.3, and 75.8% of the patients who received the 80, 160, and 240 mg doses, respectively, at week 48 and in 33.9% of the patients who received placebo. The proportion of patients achieving at least a 4-point reduction in their SELENA-SLEDAI scores at week 48 was 75.8, 77.8, and 79.0% of the patients in the telitacicept groups and 50.0% of the patients in the placebo group. Adverse events were recorded in 90.3, 92.1, 93.5, and 82.3% of the patients in the 80, 160, and 240 mg telitacicept and placebo groups, respectively. Adverse events were most commonly reactions at the injection site and infections of the upper respiratory tract. If such promising still early results are confirmed in later stage trials, telitacicept could emerge as a promising, and safe option in the management of active SLE.

### Inhibition of IFN Pathway

The story behind IFN targeting in patients with SLE is not new. More than 40 years ago it was reported that interferon is increased in the sera of patients with lupus, in active more than in inactive (31). Even though this report was about immune interferon, more recently the interest in interferons was renewed and was re-focused on IFNα and perhaps more importantly on IFN signature, based on a pivotal study by the groups of Bennett et al. (32). Anifrolumab is a fully human mAb that binds to the type I interferon receptor, blocking the activity of type I interferons such as interferon-α and interferon-β. A phase 3, randomized, double-blind, placebo-controlled trial included 362 patients with SLE. They were randomized to receive anifrolumab (*n* = 180) or placebo (*n* = 182) (33). A BICLA response was achieved in 47.8% of the patients in the anifrolumab group and 31.5% of the patients in the placebo group at week 52. For patients with a high interferon gene signature, the percentages were 48% in the anifrolumab group and 30.7% in the placebo group. For patients with a low interferon gene signature, the percentages were almost similar to those with a high interferon signature (46.7 and 35.5%, respectively). Anifrolumab also resulted in a reduction of the glucocorticoid dosages and in an improvement of skin involvement. Anifrolumab had no impressive effects in arthritis or in the annualized flare rates. Serious adverse events including pneumonia and deterioration of SLE were reported in 8.3% of the patients in the anifrolumab group and in 17% of the patients in the placebo group; one patient in the anifrolumab group died due to pneumonia. Herpes zoster infection occurred in 7.2% in the anifrolumab group and in 1.1% in the placebo group. Anifrolumab treatment also resulted in a restoration of the lymphocyte and neutrophil counts and T-cell subset counts as well as IFN-induced chemokines, such as the B-cell-targeting chemokine CXCL13/BLC, the T-cell-targeting chemokines IP10 and ITAC, as well as the levels of BAFF, CL19/MIP-3β, VCAM1, and ANGPT2, protein B2M, the soluble cofactor for TLR9 signaling, progranulin as well as MCP1 and MCP2 (34).

A meta-analysis of randomized controlled trials of mAbs targeting IFNα or type I IFN receptor subunit 1 (IFNAR) has been recently published (35). Three studies including a total of 927 patients showed that anifrolumab 300 mg was more effective than placebo in achieving SRI-4 and BICLA responses. There was also an increased risk of herpes zoster infection, nasopharyngitis, and bronchitis in 7 studies with 1,590 patients.

### Cytokines IL-12 and IL-23

Ustekinumab is a human mAb that binds the p40 subunit of IL-12 and IL-23 rendering both of them unable to bind to their receptors. Pioneering studies by Zhang and Kyttaris from the Tsokos Lab provided evidence that the IL23/IL17 axis is central in the pathogenesis of lupus nephritis in the MRL/lpr murine model (36). Double negative T cells from such mice overproduce IL17 and MRL/lpr lymph node cells, but not normal murine lymph node cells treated with IL23, transfer nephritis in non-autoimmune and lymphocyte deficient mice. A multicenter, double-blind, phase 2, randomized, controlled trial included 102 patients with active SLE (37). These patients were randomly assigned to receive ustekinumab (*n* = 60) or placebo (*n* = 42). At week 24, 37 out of 60 patients (62%) in the ustekinumab group and 14 out of 42 patients (33%) in the placebo group achieved an SRI-4 response (*p* = 0.006). Infections were reported in 45% of the patients in the ustekinumab group and in 50% of the patients in the placebo group. No deaths or malignancies were recorded in either group. Based on the encouraging results of the phase II trial, a phase III study was designed aiming to assess the efficacy and safety of ustekinumab in patients with active SLE. The manufacturer announced discontinuation of this study due to inefficacy leading to the exclusion of ustekinumab from the treatment options of SLE.

The role of IL17 was further stressed by works from LaCava Lab (38). An ongoing phase III, double-blind, placebo-controlled trial aims to evaluate the efficacy and safety of the anti-IL17 mAb secukinumab in combination with standard of care treatment in patients with active lupus nephritis (39). The primary outcome is the proportion of patients that will achieve complete renal response at week 52.

### Low Doses of IL-2

It has been suggested that low levels of IL-2 may result in disruption of immune tolerance. Lupus is a “low IL-2” disease and this is thought to play a role in the pathogenesis of the disease. According to the results of a randomized, double-blind, placebo-controlled clinical trial, low-doses of IL-2 might be a beneficial and safe choice in the treatment of patients with SLE (40). More specifically, 60 SLE patients (including patients with lupus nephritis) received either IL-2 (*n* = 30) or placebo (*n* = 30) for 12 months. The SRI-4 response rates were 55.17% in the IL-2 group and 30% in the placebo group, at week 12. At week 24, the SRI-4 response rate was 65.2% in the IL-2 group and 36.67% in the placebo group. Treatment with low doses of IL-2 was associated with a predicted expansion of peripheral Treg cells, improving perhaps immune tolerance. Addition of low-doses of IL-2 in combination with rapamycin in 50 patients with SLE resulted in a reduction of the SLEDAI score after 6, 12, and 24 weeks of treatment (41). Median prednisone dosages were decreased. The same regimen resulted in an expansion of Treg cells and a restoration of the Th17/Treg ratio declaring that Treg cells may participate in the pathogenesis of SLE. There is an ongoing trial of treatment with IL-2 at different doses in patients with SLE and its primary outcome is the SRI-4 response at week 12 (42). Studies targeting cytokines are depicted in Table 2.

**Table 2:** Regimens targeting cytokines for the treatment of SLE.

**Drug**	**Mechanism of action**	**Phase of the study**	**Manifestation**	**Primary outcome**
Belimumab	mAb that targets BLyS: inhibitis B cell survival	FDA approved	Lupus nephritis	Primary efficacy renal response
Telitacicept	TACI-Fc fusion protein that targets BLyS and APRIL: inhibits development and survival of mature B cells and plasma cells	2b	SLE	SRI-4 response at week 48
Anifrolumab	mAb that binds to IFN-I receptor: blocks the activity of type I interferons	III	SLE	BICLA response at week 52
Ustekinumab	mAb that binds to p40 subunit of IL-12 and IL-23	II	SLE	SRI-4 response at week 24
Low dose of IL-2	Restoration of immune tolerance		SLE and lupus nephritis	SRI-4 response at week 12

## Selective Inhibition of Intracellular Biochemical Pathways

### Calcineurin

Activation of the BCR and TCR in SLE is followed by an enhanced and more rapid ionized calcium influx into the cytoplasm. In T cells, Ca^2+^ activates eventually calcineurin; this effect is believed to be inhibited by calcineurin inhibitors. Voclosporin is a novel cyclosporine analog, the most potent and least toxic among all known calcineurin inhibitors. A phase 2 randomized, double-blind, placebo-controlled trial included 265 patients with lupus nephritis (43). Two doses of voclosporin (23.7 or 39.5 mg, each twice daily) were evaluated vs. placebo in combination with MMF and corticosteroids for induction of remission in lupus nephritis. The primary endpoint was complete renal remission defined as a decrease in UPCR to ≤ 0.5 in 2 consecutive measurements and an eGFR >60 ml/min per 1.73 m^2^ or no decrease of ≥20% of baseline eGFR on 2 consecutive measurements at 24 weeks. The secondary endpoint was complete renal remission at 48 weeks. Complete renal remission was achieved in 32.6% of the patients in the low dose voclosporin group, 27.3% of the patients in the high dose voclosporin group, and 19.3% of the patients in the placebo group. These data suggest that introduction of the novel calcineurin inhibitor voclosporin and specifically the low-dose regimen along with standard treatment for induction therapy of active lupus nephritis is more efficacious than MMF and corticosteroids alone. Serious adverse events were recorded in 28.1% in the low-dose regimen, in 15.9% in the placebo group and in 25% in the high-dose group. More deaths were noticed in the low-dose regimen (11.2%) compared to the high-dose regimen (2.3%) or the placebo group (1.1%). A phase 3 study showed that the addition of voclosporin to mycophenolate mofetil and low-dose corticosteroids was superior to standard treatment in patients with lupus nephritis (44). The AURORA study included 357 patients with active lupus nephritis. Renal response was achieved in 40.8% of the patients receiving voclosporin and 22.5% of those in the control group and therefore the study clearly met the primary endpoint. Patients receiving voclosporin had a 50% reduction in the UPCR faster than the control group. Serious adverse events, mainly infections were noticed in 20.8% of the patients in the voclosporin group and in 21.3% in the control group. One death was recorded in the voclosporin group and 5 deaths were reported in the control group. Renal response at 24 weeks, partial renal response at 24 and 52 weeks, time to achieve UPCR ≤ 0.5, and time for 50% reduction of UPCR were the secondary endpoints and they all displayed a statistical significance in favor of voclosporin vs. standard treatment alone. There was no significant reduction of the eGFR at week 52 in the voclosporin group or increases of glucose, lipids, or blood pressure, which are common side effects of calcineurin inhibitors. A total of 216 patients who had completed the AURORA study were enrolled into the AURORA 2, a 104-week blinded extension study in order to evaluate long-term outcomes in patients with lupus nephritis (45). Voclosporin was recently approved by the FDA as the first orally administered therapy for lupus nephritis.

### mTOR Inhibition

Sirolimus is an immunosuppressive macrolide. It blocks activation of T cells and B cells through mTOR (mammalian target of rapamycin) inhibition, reducing thereby their sensitivity to IL-2. Activation of mTOR plays a role in lupus T cell signaling dysregulation. Such mTOR-mediated lupus T cells defects were described by Fernandez et al. from the Perl Lab (46, 47). A prospective, open-label, single-arm clinical trial sirolimus was administered in 40 patients with SLE for 12 months (48). Patients with severe or life-threatening manifestations of SLE, proteinuria (an UPCR higher than 0.5) and hematological abnormalities such as anemia, leukopenia and thrombopenia had been excluded. Eleven patients discontinued the study due to lack of compliance or lack of tolerance. SLEDAI and BILAG scores were decreased in 16 out of 29 patients that completed treatment. Mean SLEDAI score was decreased from 10.2 at enrollment to 4.8 after 12 months of treatment (*p* < 0.001) and the mean BILAG score was decreased from 28.4 at enrollment to 17.4 after 12 months of treatment (*p* < 0.001). The mean daily dose of prednisone was decreased from 23.7 to 7.2 mg (*p* < 0.001) at 12 months after sirolimus initiation. Sirolimus treatment resulted in a raise of the previously reduced CD4^+^FoxP3^+^ regulatory T cells and CD8^+^ memory T cells. It also decreased the previously increased IL-4 and IL-17 production by CD4^+^ and CD3^+^CD4^−^CD8 double-negative T cells after 12 months of treatment. CD8^+^ memory T cells were selectively increased in patients with clinical improvement. Perhaps more importantly, the depletion of CD62L^−^CD197^−^ effector memory CD8^+^T cells was reportedly a predictor of a good sirolimus response.

A retrospective study included 16 patients with class III and/or V or IV and/or V or pure class V lupus nephritis who received sirolimus (49). Nine patients had intolerance to standard immunosuppressants (MMF and calcineurin inhibitors), and 7 patients had a history of cancer. Sirolimus was administered as an induction treatment in 5 and as maintenance therapy in 11 patients. Proteinuria was diminished from 2.8 ± 1.9 g/d at baseline to 0.1 ± 0.1 g/d (*p* = 0.011) at 36 months after treatment in the first group. A stable renal function was achieved in the second group. One patient experienced a renal flare and another one developed end-stage renal disease 27 months after sirolimus treatment.

A meta-analysis was conducted to determine the overall efficacy of sirolimus in patients with SLE (50). The overall reduction of SLEDAI and BILAG scores and that of corticosteroid dosages was 4.85, 1.98, and 13.17 mg/d, respectively, in 111 patients with active disease. Remission was noticed in 74% of the patients who received sirolimus for their active disease and maintenance of remission was achieved in 95.5% of the patients with lupus nephritis. Side effects were mild; only 9.3% of the patients discontinued the treatment. It is therefore plausible that mTOR inhibition may represent a promising novel approach in the treatment of patients with lupus.

### JAK-STAT Signaling

The activation of the JAK-STAT pathway plays a role in the differentiation of pathogenic effector T cells and in the impairment of Treg cells. Baricitinib is an oral inhibitor of Janus kinase (JAK), blocking the subtypes JAK1 and JAK2. In a double-blind, multicenter, randomized, placebo-controlled, 24-week phase II study, 314 patients with active SLE involving skin or joints were randomly assigned to receive placebo (*n* = 105), baricitinib 2 mg/d (*n* = 105), or baricitinib 4 mg/d (*n* = 104) (51). At week 24, reductions of SLEDAI scores were observed in 67% of the patients in the baricitinib 4 mg/d group and in 58% of the patients in the baricitinib 2 mg/d group. Baricitinib (4 mg/d), but not the lower dosage, appeared to be more effective in the management of patients with SLE that remains active despite standard treatment. Severe infections were recorded in 6% of the patients in the baricitinib 4 mg/d group, in 2% of the patients in the baricitinib 2 mg/d group and in 1% of the patients in the placebo group. Additionally, development of deep vein thrombosis was recorded in 1 patient receiving the 4 mg dosage regimen; this patient was positive for antiphospholipid antibodies. Nevertheless, the short-term follow-up of the study is insufficient to reliably determine the efficacy and safety of baricitinib in SLE.

### Antimalarials

A study of mepacrine on top of previous treatment in 46 SLE patients with refractory arthritis and/or cutaneous disease has been conducted (52). A total of 91% patients had a complete/partial response. CLASI and SLEDAI scores were significantly decreased. In addition, the mean daily dose of prednisone decreased from 5.8 to 3.4 mg/d (*p* = 0.001) and corticosteroids were finally discontinued in 20% of patients. Interestingly, smoking was the only predictor of complete response, in contrast to the doctrine that there is a reduced response of smokers to antimalarial treatment. Thus, the combination of mepacrine and hydroxychloroquine treatment could be beneficial in these patients. Interestingly, concomitant use of a combination of antimalarials did not increase retinal toxicity risk considering that the mean follow-up was 33 months. The above studies that address approaches targeting intracellular molecules and / or pathways are summarized in Table 3.

**Table 3:** Regimens targeting intracellular molecules or intracellular pathways.

**Drug**	**Mechanism of action**	**Phase of the study**	**Manifestation**	**Primary outcome**
Voclosporin	Calcineurin inhibition	FDA approved	Lupus nephritis	Complete renal response
Sirolimus	mTOR inhibition	1/2	SLE	Decrease in SLEDAI and BILAG scores at each visit (months 1–12)
		Retrospective	Lupus nephritis	
Baricitinib	JAK1 and JAK2 inhibitor	II	SLE	SLEDAI-2K at week 24
Mepacrine	Unknown	Retrospective analysis of the prospectively acquired data	SLE	

## Ongoing Clinical Trials

Apart from trials that were mentioned above, other ongoing clinical studies can be grouped as follows:

### Fighting T Cells

T cells are essential players in the autoimmune response of lupus patients. Dapirolizumab pegol is an anti-CD40L pegylated Fab fragment that blocks costimulatory interactions between T cells and antigen presenting cells expressing CD40. A phase 2b study of dapirolizumab pegol in patients with active SLE with an inadequate response to standard treatment has been carried out (53). The study did not meet its primary endpoint (achieving a dose-response at 24 weeks). SLEDAI and PGA did not differentiate treatment groups; changes of BICLA and SRI-4 scores were assigned to escape medicines given during the study.

Several years ago, a trial of another mAb against CD40L in patients with lupus nephritis was terminated prematurely (54). However, the costimulatory pathway initiated by CD40L still remains an attractive target in SLE and therefore investigators continue the efforts in order to determine the efficacy of dapirolizumab (a pegylated construct and not a full antibody) in a phase III study (55). The primary outcome is BICLA response at week 48.

Itolizumab (EQ001) is a monoclonal antibody targeting the CD6 receptor on the surface of T cells. It blocks the binding of CD6 on its ALCAM (activated leukocyte cell adhesion molecule) ligand, inhibiting therefore immune responses mediated by T cells. Data were presented at the 2019 ACR/ARP Annual Meeting (56). CD6 and ALCAM positive cells were reportedly increased in patients with lupus nephritis and were associated with SLE activity. Increased excreted ALCAM levels were also measured in the urine of patients with active lupus nephritis. Itolizumab ameliorated renal disease in murine models, decreased the migration of T cells to inflamed tissues and also increased levels of IL-10. In addition, itolizumab resulted in suppression of T-cell development and proliferation. Based on animal model data, the manufacturer was granted a U.S. FDA fast-track designation for itolizumab for the treatment of lupus nephritis. The EQUALIZE trial is designed to include 2 groups. The first group is composed of patients with SLE that will receive itolizumab subcutaneously every 2 weeks for 4 weeks, while the second group consists of patients with lupus nephritis to receive itolizumab or placebo for 12 weeks.

LY3471851 (NKTR-358) is a novel Treg cell stimulator through targeting the IL-2 receptor complex. It is designed to correct specifically this immune system abnormality, i.e., the deficiency in Treg in patients with lupus, and it does not affect the entire immune system. The primary outcome of a phase 2 study is the percentage of patients that will achieve a ≥4-point reduction in SLEDAI-2K Score at week 24 (57). Despite the non-encouraging results of previous attempts in T cell costimulation blockade in patients with SLE, a phase 2 study aims to assess the efficacy of abatacept in patients with SLE and the primary endpoint is the BICLA response at 6 months (58).

### Targeting B Cells and Beyond

B cells are being targeted directly or indirectly in patients with lupus. RC18 is a recombinant human BLyS receptor antibody fusion protein and it is used in a phase III placebo-controlled study plus standard treatment with primary outcome an SRI response rate at week 52 (59). CC-220 is a cereblon modulator causing potent degradation of Ikaros and Aiolos leading to suppressed B cell proliferation and cytokine production. A phase 2, placebo-controlled study aims to evaluate efficacy and safety of CC-220 in patients with active SLE and the primary outcome is an SRI-4 at week 24 (60). B cell and T cell collaboration is essential for the lupus autoimmune response. To this end, AMG 570, an ICOSL and BAFF bispecific inhibitory antibody, has been employed in a phase 2b study. The primary endpoint is the percentage of patients achieving an SRI-4 at week 52 (61). Based on the same concept, VAY736 or Ianalumab, a mAb that blocks the BAFF receptor and CFZ533 or iscalimab, a mAb that prevents CD40 pathway signaling are under investigation in a phase 2 study in patients with SLE with a primary outcome of an SRI-4 response at week 29 (62).

BTK inhibitors, JAK inhibitors, and some other agents with different targets are also currently under investigation and are summarized in Table 4.

**Table 4:** BTK inhibitors, JAK inhibitors, and other agents that are currently under investigation.

**BTK inhibitors**	**JAK inhibitors**	**Miscellaneous**
Fenebrutinib (GDC-0853) (63)	Upadacitinib (JAK1 inhibitor) (64)	Lenabasum (JBT-101) (endocannabinoid type 2 receptor agonist) (65)
Orelabrutinib (ICP-022) (66)	Tofacitinib (JAK1, JAK3, JAK2 inhibitor) (67)	Memantine (NMDA receptor antagonist) (68)
Branebrutinib (69)	PF-06700841 or Brepocitinib (JAK1, TYK2 inhibitor) (70)	EBV-specific cytotoxic T lymphocytes (71)
Elsubrutinib (64)	BMS-986165 or Deucravacitinib (TYK2 inhibitor) (72)	Mesenchymal stem cells in A) SLE (73) B) Lupus nephritis (74)
		Curcumin (75)

## Other Potential Therapeutic Targets

Experimental animal studies have examined microglial-targeted therapies in neuropsychiatric SLE (NPSLE) (76). Agents aiming to the treatment of NPSLE are seriously lacking from our therapeutic armamentarium. Fingolimod, an S1P receptor modulator, resulted in improvement of NPSLE-like manifestations in mice such as depressive-like behavior and memory deficits. Fingolimod has been already approved for the treatment of patients with multiple sclerosis and previous studies could impel the potential use of this agent in the management of NPSLE patients. Similarly, angiotensin-converting enzyme inhibitors suppress microglial activation and they could also be considered in such patients, aiming to treat cognitive dysfunction (77).

Cenerimod is a selective agonist for the G-protein-coupled sphingosine-1-phosphate receptor 1 (S1P receptor 1 or S1P1), also known as endothelial differentiation gene 1 (EDG1). It is a potent immunomodulator due to its effects in the number of circulating and infiltrating T- and B-cells. In a phase II study, patients with SLE received cenerimod treatment at different doses (78). T- and B-cells were measured by flow cytometry before and after 12 weeks of treatment. According to the results, there was a reduction of CD4^+^ T cells (95%) and CD19^+^ B cells (90%) and also a reduction of antibody-secreting cells (85%). No information on the safety of this agent are known.

A novel, orally given, small molecule inhibitor, R835, that targets IRAK-1, and IRAK-4- mediated signaling would expand the therapeutic options in SLE by aiming at molecules of the innate immunity pathway and/or innate immune cells. Administration of R835 in NZB/W F1 lupus-prone mice ameliorates their renal disease (79). Inhibition of IRAK1 and IRAK4 kinases suppress TLR and IL-1R signaling and the subsequent production of pro-inflammatory cytokines.

## Discussion

**Figure 1:**
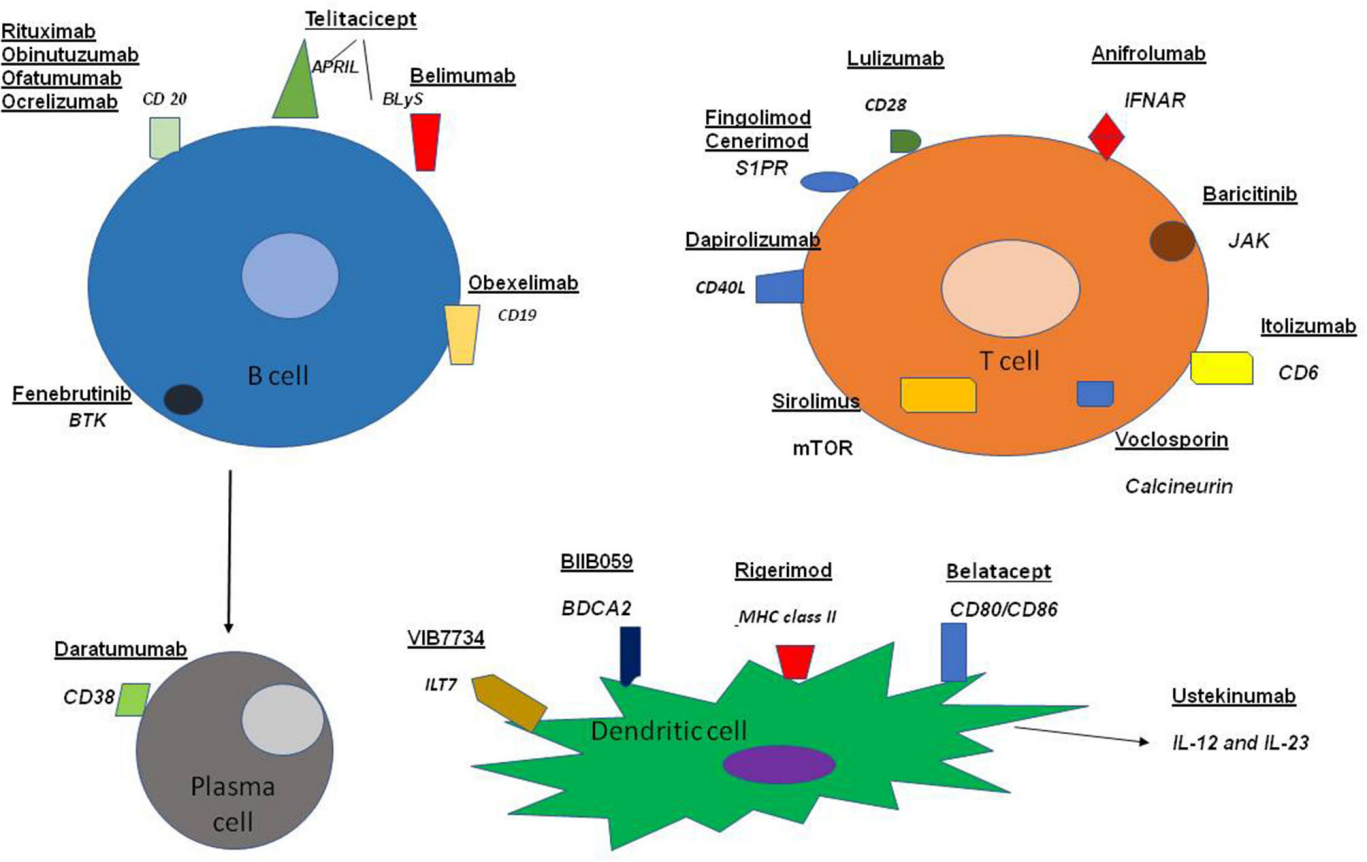
Molecules targeted therapeutically in patients with SLE.

Our review highlights ongoing efforts dealing with the management of SLE. The trials that have been carried out, or are currently under way, include a variety of agents in view of the diversity of the disturbances of the immune system encountered in patients with SLE and are diagrammatically depicted in Figure 1.

It might be tricky to attempt to explain the reason(s) for the failure of some regimens and for the success of some others. B cell qualitative and quantitative abnormalities are the hallmark in the pathogenesis of SLE. B cell targeting therapies seem to achieve better clinical responses than treatments targeting T cells. However, the large clinical trials of RTX failed to meet their primary endpoint. It has been hypothesized that the reason was the inappropriate design of the studies, whereas others suggested that B cell depletion was insufficient. Regarding trial design, the large approval studies of belimumab altered their primary outcome with the agreement of the relevant regulatory authorities, in order to achieve more feasible, yet clinically meaningful results. Therefore, the SRI-4 response was introduced. Another example of adjusting the trials' design is the following: the second phase III trial of anifrolumab changed its primary outcome toward a secondary endpoint previously employed in another study that had failed.

Focusing on the issue of the potentially insufficient B cell depletion, obinutuzumab was tested in lupus nephritis patients verifying investigators expectations (5). It was highlighted that efficient B cell depletion was clearly associated with the long-standing beneficial effects of obinutuzumab in lupus nephritis patients (6). Additionally, potential concerns regarding its safety were defeated due to a lower rate of adverse events in the obinutuzumab group when compared with the placebo group.

Lupus nephritis is an aspect of the disease often difficult to treat. Fortunately, two drugs, the orally given voclosporin and the intravenous form of belimumab, have recently been approved from the FDA for the treatment of patients with lupus nephritis on top of standard of care. Another recent report suggests daratumumab, targeting long-lived plasma cells (as well as other cells previously mentioned), as an alternative therapeutic approach in SLE (11). Daratumumab induced remission in 2 patients with life threatening manifestations including lupus nephritis. However, studies with meaningfully larger groups of SLE patients are necessary to determine the efficacy and safety of daratumumab in lupus. A pilot study suggests that the mTOR inhibitor sirolimus could also be a generally safe and an alternative option in the management of lupus nephritis in patients who are intolerant to standard therapy or in cases of a history of malignancy (31). Treatment options of NPSLE, another severe manifestation of SLE, remain poor. Even fatigue, a common symptom decreasing the quality of patients' life, cannot be managed sufficiently so far. There is an evolving landscape of SLE treatments from agents with multiple, non-specific targets such as glucocorticoids and cyclophosphamide to selective treatments. Current approaches specifically target cytokines (e.g., BLyS), intracellular pathways (e.g., calcineurin) as well as cell populations (e.g., B cells) according to the advances in our understanding of the pathogenesis of SLE. Sometimes a combination of treatments might be necessary given the fact that lupus is a multifactorial disease. Moreover, because the long-standing clinical knowledge that no 2 lupus patients are identical is true, personalized approaches might also be important.

Taken together, the results of several studies are at least encouraging but none of them has emerged as a “panacea” for SLE. A current, reasonably attractive target of treatment in SLE would be the autoreactive B cells specifically, and not the total of the B cell population. However, the only approach that provided evidence of specific annihilation of the autoreactive B cell pool, belimumab following RTX, was not clinically much effective. Generalized immunosuppression should be minimized with the introduction of novel agents since infections, potentially life threatening, are always an important issue.

## Author Contributions

SL: paper concept and wrote the paper. CS: wrote the paper. Both authors contributed to the article and approved the submitted version.

## Conflict of Interest

The authors declare that the research was conducted in the absence of any commercial or financial relationships that could be construed as a potential conflict of interest.
